# Supplementary material for: ProtoCloud: A prototypical self-explaining model for single-cell analysis
Source: Cell Genom. 2026 Apr 16;6(6):101217. doi: 10.1016/j.xgen.2026.101217 (PMC13261663; doi:10.1016/j.xgen.2026.101217)
Supplement: Document S1. Figures S1–S6 and Tables S1–S7 [file mmc1.pdf]

Cell Genomics, Volume *6*

Supplemental information

ProtoCloud: A prototypical self-explaining model for single-cell analysis

Kaiyun Guo and Jiarui Ding

Document S1. Supplementary Information

Contents: Figures S1–S6, Tables S1–S7.

Supplementary Figures

Figure S1 . . . 3
Figure S2 . . . 4
Figure S3 . . . 5
Figure S4 . . . 7
Figure S5 . . . 9
Figure S6 . . . 11

Supplementary Tables

Table S1 . . . 12
Table S2 . . . 12
Table S3 . . . 12
Table S4 . . . 13
Table S5 . . . 13
Table S6 . . . 13
Table S7 . . . 14

Supplementary Figures

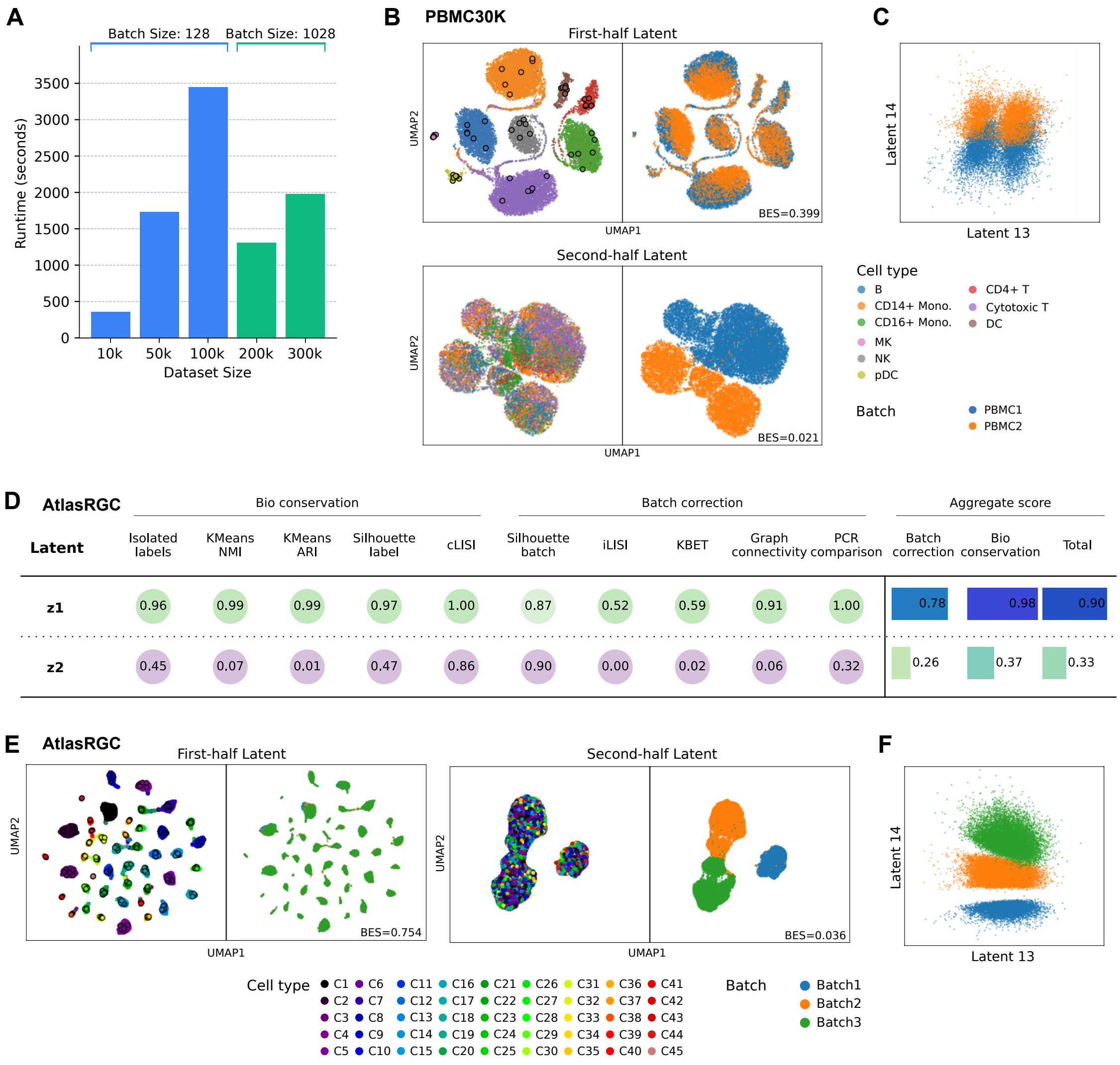

Figure S1: ProtoCloud runtime and visualization of latent embeddings, related to Figure 3

(A) Training time of ProtoCloud versus data size. All experiments were conducted on a server with one NVIDIA Tesla V100 16GB GPU on a single node. Training time scales linearly with dataset size, with smaller datasets processing efficiently and larger ones benefiting from an increased batch size.
(B) UMAP visualization of the PBMC30K[1] dataset. Left: colored by cell type. Right: colored by batch. Prototypes are shown as dots with black edges.
(C) Direct visualization of PBMC30K latent dimensions 13 and 14.
(D) Evaluation of latent space disentanglement using the AtlasRGC[2] dataset. Comparison of scIB metrics for the biological subspace (first-half latent space, $\mathbf{z}^1$) and the batch subspace (second-half latent space, $\mathbf{z}^2$). Metrics are grouped into biological conservation and batch correction categories.
(E) UMAP visualization of AtlasRGC.
(F) Direct visualization of AtlasRGC latent dimensions 13 and 14.

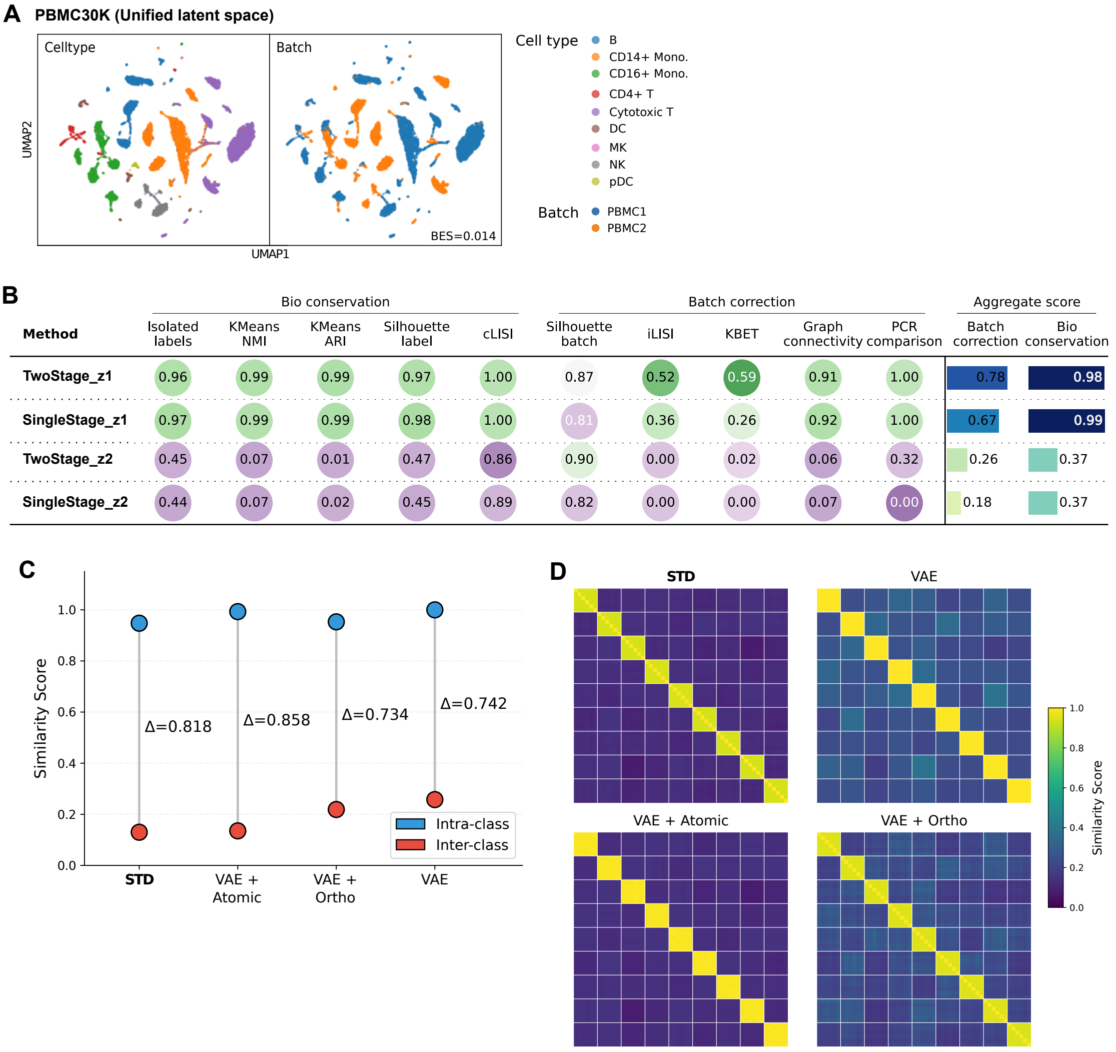

Figure S2: Ablation study of ProtoCloud model components, related to Figure 2-3

(A) UMAP visualization of ProtoCloud with unified latent space in the PBMC30K dataset, colored by cell type (left) and batch (right).

(B) Comparison of scIB metrics between two-stage curriculum and single-stage end-to-end training.

(C) Effect of loss components on prototype separation. Pairwise similarities are computed among prototypes, with intra-class similarity measured between prototypes of the same cell type and inter-class similarity between prototypes of different cell types. The four configurations are: standard ProtoCloud (STD), VAE with atomic loss only (VAE + Atomic), VAE with orthogonal loss only (VAE + Ortho), and VAE without orthogonal and atomic losses (VAE). △ indicates the difference between intra-class and inter-class similarity.

(D) Heatmaps of pairwise prototype similarity for each loss configuration.

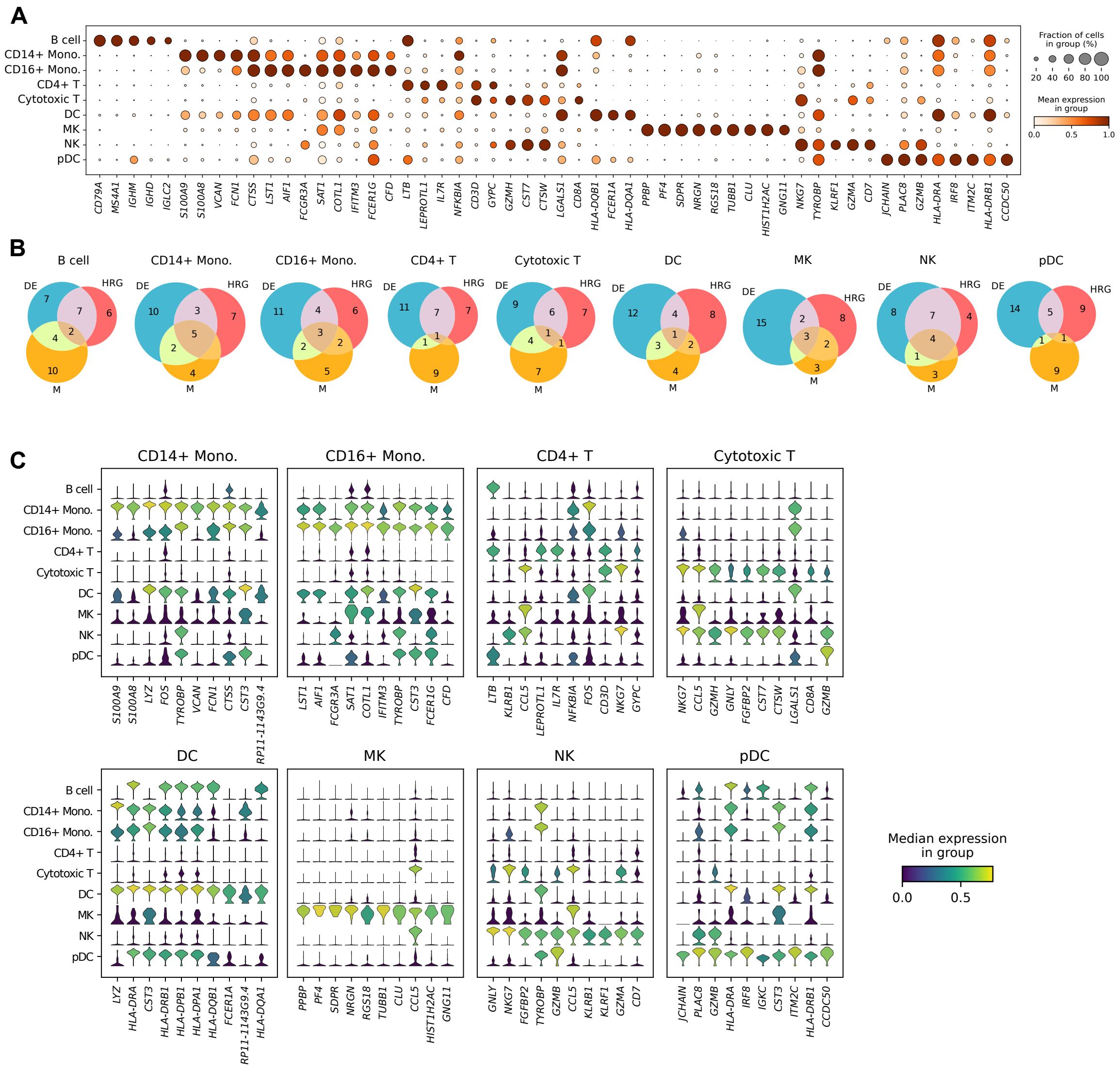

Figure S3: Highly relevant genes in the PBMC30K dataset[1], related to Figure 3

(A) Dot plot of top ranked HRGs across cell types in PBMC30K. The gene set includes the top 10 HRGs for each cell type, where duplicated genes were removed to ensure a unique gene set.

(B) Overlap of differentially expressed genes (DE), highly relevant genes (HRG), and canonical marker genes (M) for each cell type in PBMC30K. The overlap is shown with the top 20 DE genes, top 15 HRGs, and known markers (10-16 per type).

(C) Violin plot of top HRGs for each cell type in PBMC30K. Each subplot highlights the distribution of the top ten HRG expression across different cell types.

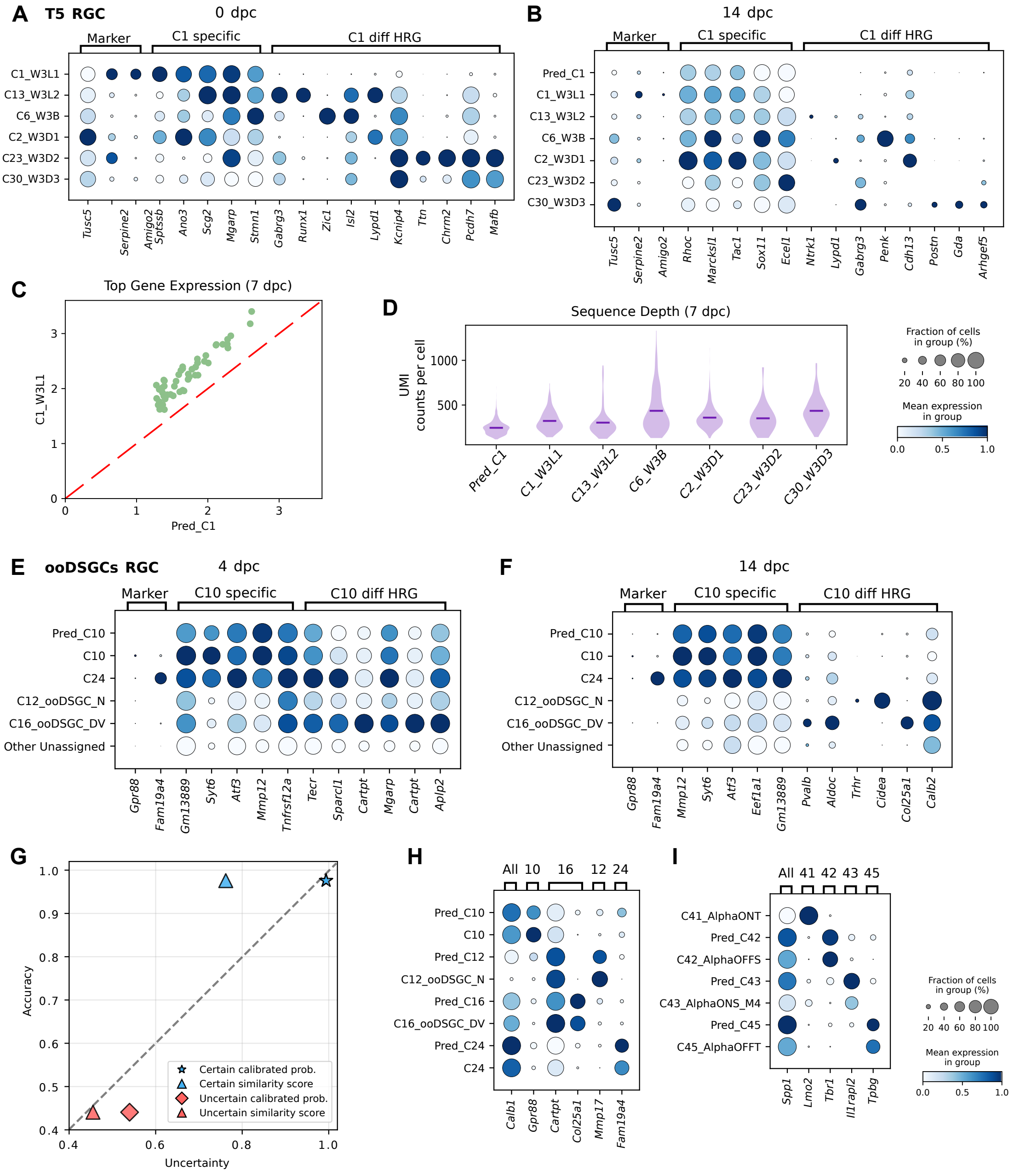

A
T5 RGC
0 dpc
Marker
C1 specific
C1 diff HRG
C1_W3L1
C13_W3L2
C6_W3B
C2_W3D1
C23_W3D2
C30_W3D3
Tusc5
Serpine2
Amigo2
Sptssb
Ano3
Scg2
Mgarp
Stmn1
Gabrg3
Runx1
Zic1
Isl2
Lypd1
Kcnip4
Ttn
Chrm2
Pcdh7
Mafb
B
14 dpc
Marker
C1 specific
C1 diff HRG
Pred_C1
C1_W3L1
C13_W3L2
C6_W3B
C2_W3D1
C23_W3D2
C30_W3D3
Tusc5
Serpine2
Amigo2
Rhoc
Marcksl1
Tac1
Sox11
Ecel1
Ntrk1
Lypd1
Gabrg3
Penk
Cdh13
Postn
Gda
Arhgef5
C
Top Gene Expression (7 dpc)
C1_W3L1
Pred_C1
D
Sequence Depth (7 dpc)
UMI
counts per cell
1000
500
Pred_C1
C1_W3L1
C13_W3L2
C6_W3B
C2_W3D1
C23_W3D2
C30_W3D3
Fraction of cells
in group (%)
20 40 60 80 100
Mean expression
in group
0.0 0.5 1.0
E
ooDSGCs RGC
4 dpc
Marker
C10 specific
C10 diff HRG
Pred_C10
C10
C24
C12_ooDSGC_N
C16_ooDSGC_DV
Other Unassigned
Gpr88
Fam19a4
Gm13889
Syt6
Atf3
Mmp12
Tnfrsf12a
Tecr
Sparcl1
Cartpt
Mgarp
Cartpt
Aplp2
F
14 dpc
Marker
C10 specific
C10 diff HRG
Pred_C10
C10
C24
C12_ooDSGC_N
C16_ooDSGC_DV
Other Unassigned
Gpr88
Fam19a4
Mmp12
Syt6
Atf3
Eef1a1
Gm13889
Pvalb
Aldoc
Trhr
Cidea
Col25a1
Calb2
G
Accuracy
Uncertainty
Certain calibrated prob.
Certain similarity score
Uncertain calibrated prob.
Uncertain similarity score
H
All 10 16 12 24
Pred_C10
C10
Pred_C12
C12_ooDSGC_N
Pred_C16
C16_ooDSGC_DV
Pred_C24
C24
Calb1
Gpr88
Cartpt
Col25a1
Mmp17
Fam19a4
I
All 41 42 43 45
C41_AlphaONT
Pred_C42
C42_AlphaOFFS
Pred_C43
C43_AlphaONS_M4
Pred_C45
C45_AlphaOFFT
Spp1
Lmo2
Tbr1
Il1rapl2
Tpbg
Fraction of cells
in group (%)
20 40 60 80 100
Mean expression
in group
0.0 0.5 1.0

Figure S4: Characterization of predicted "unassigned" cells in comparison with reference RGC subtypes, related to Figure 5

(A)-(B) Comparison of HRG expression between W3-like C1 RGCs and other T5 RGCs, including unassigned cells predicted as C1 (Pred_C1) cells. Dot plots showing the expression patterns of representative genes across different RGC subtypes at 0 (**A**) and 14 (**B**) days post-crush (dpc). Genes include known marker genes (*Tusc5*, *Serpine2*, and *Amigo2*), HRGs specific to C1 RGCs (C1 specific) and differentially ranked HRGs between C1 and other T5 RGCs.

(C) The expression of the top highly expressed genes in predicted and reference C1 cells at 7 dpc. Points above the diagonal red dashed line indicate a systematic downregulation of these genes in predicted C1 cells compared to reference cells.

(D) Comparison of UMI counts between predicted C1 cells (Pred_C1) and reference T5 RGC subtype populations at 7 dpc. Violin plots show the distribution of sequencing depth, with horizontal lines marking the mean.

(E)-(F) Comparison of HRG expression between C10 RGCs and other ooDSGCs RGCs, including unassigned cells predicted as C10 (Pred_C10) cells. Dot plots showing the expression patterns of representative genes across different RGC subtypes at 4 dpc (**E**) and 14 dpc (**F**).

(G) Reliability diagram of Patch-seq RGC certainty estimates. Both the model and the calibrator were trained on the AtlasRGC dataset and subsequently applied to Patch-seq RGC predictions. The plot compares uncertainty estimates derived from similarity scores and calibrated similarities for both certain and uncertain groups.

(H-I) Validation of transferred annotations on Patch-seq RGC data. The dot plots compare the expression of marker genes between the most abundant predicted Patch-seq cells and their matched subtypes in AtlasRGC references for S2/S4 laminating RGCs (**H**) and α RGCs (**I**).

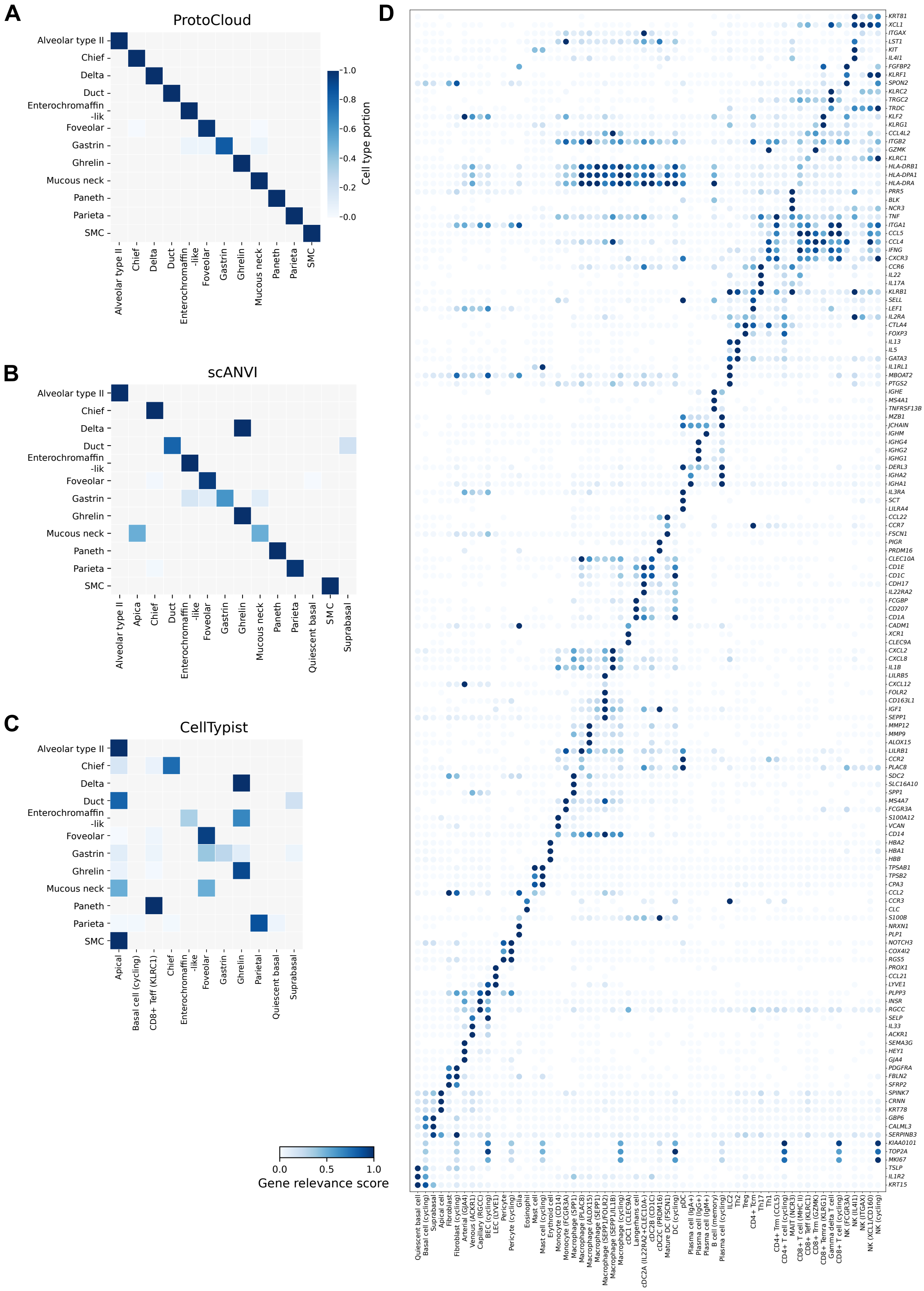
A
ProtoCloud
Alveolar type II
Chief
Delta
Duct
Enterochromaffin
-lik
Foveolar
Gastrin
Ghrelin
Mucous neck
Paneth
Parieta
SMC
Cell type portion
1.0
0.8
0.6
0.4
0.2
0.0
B
scANVI
Apica
Quiescent basal
Suprabasal
C
CellTypist
Apical
Basal cell (cycling)
CD8+ Teff (KLRC1)
Parietal
D
Gene relevance score
0.0
0.5
1.0

Figure S5: Model performance on AtlasEoE, related to Figure 6

(A-C) Confusion matrix of rare cell types in AtlasEoE[3] across **(A)** ProtoCloud, **(B)** scANVI, and **(C)** CellTypist.
(D) AtlasEoE marker genes relevance score dot plot. The dot plot illustrates the relevance scores of the provided marker genes (columns) for each of the 60 prevalent cell subsets (rows). This representation is adapted from the original study[3], which used mean gene expression levels to represent marker gene–cell type relationships. The similarity in overall cell–gene relationships between the two approaches suggests that our relevance scores reliably capture the same biological trends as those reported in the original study.

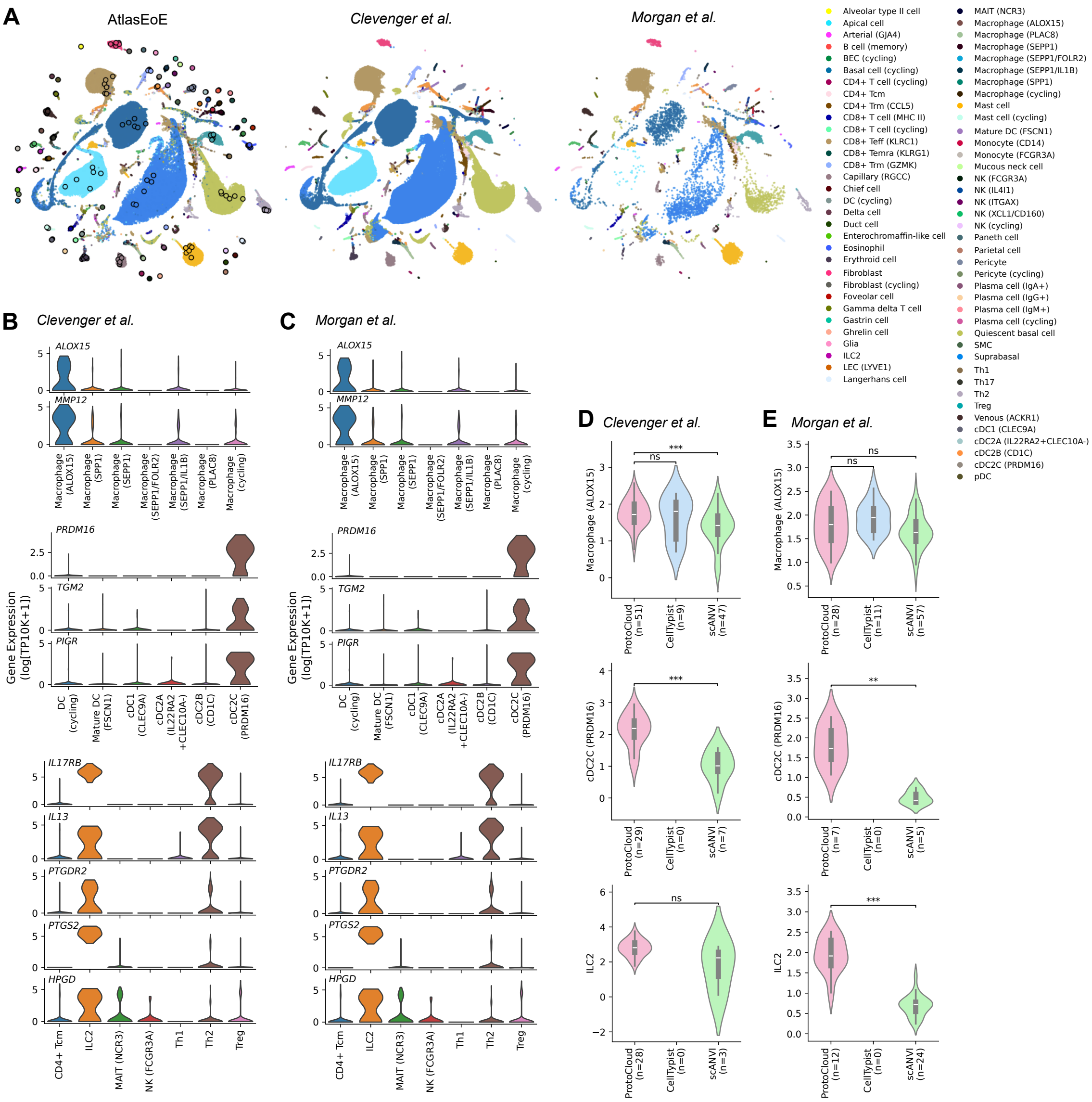
A
AtlasEoE
Clevenger et al.
Morgan et al.
B
C
D
E
Gene Expression (log[TP10K+1])
ALOX15
MMP12
PRDM16
TGM2
PIGR
IL17RB
IL13
PTGDR2
PTGS2
HPGD
Macrophage (ALOX15)
cDC2C (PRDM16)
ILC2
ProtoCloud
CellTypist
scANVI
ns
***
**

Figure S6: Model performance on applied esophageal tissue datasets, related to Figure 6

(A) UMAP visualization of the latent space embeddings for the AtlasEoE[3] (left), Clevenger et al.[4] (middle), and Morgan et al.[5] (right) datasets.

(B) Marker gene expression across cell types annotated by ProtoCloud in the Clevenger et al.[4] dataset. Expression of marker genes *ALOX15* and *MMP12* in macrophage populations (top), *PRDM16*, *TGM2*, and *PIGR* expression across dendritic cell populations (middle), and ILC2 marker expression across several rare immune cell populations (bottom).

(C) Corresponding marker expression patterns in the Morgan et al.[5] dataset.

(D) Specificity assessment of cell predictions in the Clevenger et al. dataset. Violin plots display the distribution of lineage-specific gene signature scores for *ALOX15*$^{+}$ macrophages (top), *PRDM16*$^{+}$ DCs (middle), and ILC2s (bottom). The plots compare the population identified by ProtoCloud (baseline) with non-overlapping cells identified exclusively by CellTypist or scANVI (n indicates the number of cells). Signature scores were calculated based on the top 30 marker genes derived from AtlasEoE. Box plots indicate the median, interquartile range (25th–75th percentiles), and whiskers extending 1.5× the interquartile range from the box limits. Statistical significance was assessed using two-sided Mann-Whitney U tests comparing each method against ProtoCloud (ns: non-significant, ** $p \leq 0.01$, *** $p \leq 0.001$).

(E) Corresponding specificity assessment of cell predictions in the Morgan et al. dataset.

Supplementary Tables

**Dataset**	**ProtoCloud**	Seurat	scANVI	CellTypist	scPoli	TOSICA	SIMS	scGPT	scBERT
PBMC10K	**0.969** $\pm$ **0.003**	0.977 $\pm$ 0.004*	0.973 $\pm$ 0.004**	0.952 $\pm$ 0.003**	0.935 $\pm$ 0.010**	0.967 $\pm$ 0.004	0.643 $\pm$ 0.039**	0.856 $\pm$ 0.007**	0.930 $\pm$ 0.034
PBMC30K	**0.930** $\pm$ **0.005**	0.942 $\pm$ 0.003*	0.932 $\pm$ 0.003	0.917 $\pm$ 0.003**	0.892 $\pm$ 0.004**	0.912 $\pm$ 0.008*	0.569 $\pm$ 0.032**	0.804 $\pm$ 0.007**	0.894 $\pm$ 0.025*
AtlasRGC	**0.979** $\pm$ **0.002**	0.968 $\pm$ 0.002**	0.976 $\pm$ 0.001*	0.974 $\pm$ 0.002**	0.943 $\pm$ 0.004**	0.942 $\pm$ 0.010**	0.287 $\pm$ 0.042**	0.232 $\pm$ 0.004**	0.973 $\pm$ 0.001**
TSCA_lung	**0.946** $\pm$ **0.001**	0.927 $\pm$ 0.002**	0.945 $\pm$ 0.002	0.927 $\pm$ 0.003**	0.852 $\pm$ 0.007**	0.915 $\pm$ 0.005**	0.816 $\pm$ 0.023**	0.624 $\pm$ 0.077**	0.912 $\pm$ 0.019*
TSCA_oesophagus	**0.961** $\pm$ **0.002**	0.951 $\pm$ 0.002**	0.961 $\pm$ 0.001	0.938 $\pm$ 0.002**	0.872 $\pm$ 0.005**	0.942 $\pm$ 0.002**	0.838 $\pm$ 0.121	0.811 $\pm$ 0.002**	0.945 $\pm$ 0.005**
TSCA_spleen	**0.891** $\pm$ **0.005**	0.857 $\pm$ 0.002**	0.895 $\pm$ 0.003	0.865 $\pm$ 0.004**	0.760 $\pm$ 0.005**	0.830 $\pm$ 0.004**	0.514 $\pm$ 0.064**	0.608 $\pm$ 0.004**	/
AtlasEoE	**0.945** $\pm$ **0.002**	0.916 $\pm$ 0.001**	0.951 $\pm$ 0.001**	0.925 $\pm$ 0.001**	0.800 $\pm$ 0.007**	0.927 $\pm$ 0.002**	0.709 $\pm$ 0.095**	0.584 $\pm$ 0.083**	/
ICA	**0.912** $\pm$ **0.002**	0.879 $\pm$ 0.002**	0.920 $\pm$ 0.001**	0.891 $\pm$ 0.001**	0.627 $\pm$ 0.003**	0.844 $\pm$ 0.002**	0.386 $\pm$ 0.029**	0.537 $\pm$ 0.096**	/

Table S1: Classification accuracy comparison between ProtoCloud and benchmark methods across eight datasets, related to Figure 2 ProtoCloud results are shown as mean $\pm$ standard error from five independent runs with different random seeds. Statistical comparisons between each benchmark method and ProtoCloud were performed using two-sided paired t-tests, with p-values adjusted for multiple comparisons using Benjamini-Hochberg (FDR) correction ($^{*}\ p < 0.05$, $^{**}\ p < 0.01$). "/" denotes methods that failed to run on the corresponding dataset.

**Dataset**	**ProtoCloud**	Seurat	scANVI	CellTypist	scPoli	TOSICA	SIMS	scGPT	scBERT
PBMC10K	**0.930** $\pm$ **0.016**	0.966 $\pm$ 0.009*	0.938 $\pm$ 0.012	0.902 $\pm$ 0.022*	0.902 $\pm$ 0.016*	0.941 $\pm$ 0.006	0.264 $\pm$ 0.028**	0.574 $\pm$ 0.013**	0.772 $\pm$ 0.149
PBMC30K	**0.917** $\pm$ **0.016**	0.925 $\pm$ 0.011	0.912 $\pm$ 0.019	0.879 $\pm$ 0.028	0.847 $\pm$ 0.023**	0.871 $\pm$ 0.040	0.278 $\pm$ 0.038**	0.531 $\pm$ 0.037**	0.726 $\pm$ 0.127
AtlasRGC	**0.969** $\pm$ **0.002**	0.933 $\pm$ 0.009**	0.966 $\pm$ 0.002	0.954 $\pm$ 0.006**	0.903 $\pm$ 0.005**	0.929 $\pm$ 0.014**	0.131 $\pm$ 0.039**	0.091 $\pm$ 0.004**	0.956 $\pm$ 0.005**
TSCA_lung	**0.924** $\pm$ **0.005**	0.858 $\pm$ 0.004**	0.911 $\pm$ 0.010*	0.853 $\pm$ 0.006**	0.757 $\pm$ 0.010**	0.866 $\pm$ 0.014**	0.453 $\pm$ 0.031**	0.238 $\pm$ 0.048**	0.804 $\pm$ 0.058**
TSCA_oesophagus	**0.955** $\pm$ **0.004**	0.828 $\pm$ 0.017**	0.942 $\pm$ 0.003**	0.845 $\pm$ 0.019**	0.676 $\pm$ 0.030**	0.923 $\pm$ 0.006**	0.299 $\pm$ 0.144**	0.168 $\pm$ 0.002**	0.805 $\pm$ 0.118*
TSCA_spleen	**0.862** $\pm$ **0.004**	0.762 $\pm$ 0.005**	0.856 $\pm$ 0.003*	0.799 $\pm$ 0.005**	0.653 $\pm$ 0.013**	0.783 $\pm$ 0.007**	0.226 $\pm$ 0.046**	0.235 $\pm$ 0.006**	/
AtlasEoE	**0.877** $\pm$ **0.011**	0.549 $\pm$ 0.015**	0.841 $\pm$ 0.005**	0.604 $\pm$ 0.009**	0.397 $\pm$ 0.011**	0.794 $\pm$ 0.007**	0.100 $\pm$ 0.023**	0.069 $\pm$ 0.016**	/
ICA	**0.888** $\pm$ **0.005**	0.823 $\pm$ 0.009**	0.889 $\pm$ 0.005	0.758 $\pm$ 0.009**	0.524 $\pm$ 0.003**	0.810 $\pm$ 0.005**	0.079 $\pm$ 0.017**	0.134 $\pm$ 0.036**	/

Table S2: Classification macro F1 comparison between ProtoCloud and benchmark methods across eight datasets, related to Figure 2 ProtoCloud results are shown as mean $\pm$ standard error from five independent runs with different random seeds. Statistical comparisons between each benchmark method and ProtoCloud were performed using two-sided paired t-tests, with p-values adjusted for multiple comparisons using Benjamini-Hochberg (FDR) correction ($^{*}\ p < 0.05$, $^{**}\ p < 0.01$). "/" denotes methods that failed to run on the corresponding dataset.

**Gene**	**Dataset**		
	Lung	Spleen	Esophagus
EEF1A1	1	2	3
CD74	2	1	4
HLA-DRA	3	5	6
B2M	4	4	2
IGKC	5	10	5
MS4A1	6	30	41
TPT1	7	7	9
FTH1	8	22	15
CD79A	9	24	39
HLA-B	10	19	12

Table S3: Rankings of learned HRGs for mature B cells from datasets of different organs, related to Figure 3 The three datasets used were from the lung, spleen, and esophagus of the Tissue Stability Cell Atlas. Because the esophagus dataset does not contain a mature B cell class, *CD27*$^{+}$ B cells were used instead.

DPC	Cells	ProtoCloud				CellTypist				scANVI
		Acc.	Certain (%)	Certain Acc.	Amb. Acc.	Acc.	Certain (%)	Certain Acc.	Amb. Acc.	Acc.
0 (ctrl)	12062	0.949	73.1%	0.992	0.834	**0.955**	72.3%	0.985	0.877	0.950
0.5	13619	0.938	78.4%	0.988	0.759	**0.944**	57.2%	0.988	0.886	0.943
1	12478	**0.921**	86.6%	0.964	0.641	0.906	38.3%	0.989	0.855	0.904
2	10695	**0.863**	83.7%	0.929	0.524	0.837	15.1%	0.973	0.812	0.838
4	10599	**0.656**	69.1%	0.766	0.410	0.483	2.8%	0.626	0.479	0.533
7	8700	**0.627**	74.5%	0.717	0.365	0.399	4.8%	0.581	0.390	0.506
14	8456	**0.685**	82.6%	0.748	0.387	0.396	4.7%	0.802	0.376	0.616

Table S4: Iterative training and evaluation on time-course RGC ONC dataset split by time post-crush, related to Figure 5 Only labeled cells are included in the evaluation. The ProtoCloud accuracies were computed from the final predictions after continue-training. CellTypist was initially trained on the control data (0 dpc) and used to predict labels for the data from 0.5 dpc. The confidently predicted 0.5 dpc cells (confidence score > 0.5) were then added to the training set, and the process was repeated for the data from each subsequent time point, progressively expanding the training set with predicted labels from earlier stages. scANVI followed the same incremental training procedure, but included all predicted cells during continue training as the model does not provide confidence estimates.

DPC	Cells	"Unknown" cells	Certain "unknown" (%)	
			ProtoCloud	CellTypist
0 (ctrl)	12062	0	/	/
0.5	13619	157	22.3%	56.6%
1	12478	370	42.2%	37.2%
2	10695	501	48.5%	14.4%
4	10599	2522	62.5%	4.1%
7	8700	2178	72.4%	8.2%
14	8456	1455	73.6%	4.8%

Table S5: Percentage of confidently labeled previously unknown cells at varying DPC concentrations, related to Figure 5 Shown are the number of total cells, previously "unknown" cells across different time points, and the percentage of these previously unknown cells that were confidently identified by ProtoCloud and CellTypist. Over the time course, approximately 20% to 70% of previously unknown cells were confidently assigned to a cell type by ProtoCloud.

Gene	ILC2 Rel	ILC2 Rank	T_H2 Rel	T_H2 Rank
KLRB1	0.760	1	0.474	3
IL32	0.565	2	0.529	1
ALOX5AP	0.404	3	0.505	2
IL13	0.399	4	0.294	5
PTGS2	0.273	5	0.002	633
GATA3	0.259	6	0.226	8
SRGN	0.252	7	0.333	4
HPGDS	0.248	8	0.185	13
VIM	0.243	9	0.101	23
BIRC3	0.211	10	0.081	32
IL17RB	0.196	11	0.227	7
FTH1	0.174	12	0.189	12
CD69	0.167	13	0.036	74
AREG	0.162	14	0.012	177
TRDC	0.155	15	0.001	700
SAMSN1	0.141	16	0.117	19
PTGDR2	0.140	17	0.016	148
NFKBIA	0.138	18	0.019	128
TNFRSF18	0.130	19	0.026	97
JUN	0.130	20	0.014	164

Table S6: Top ILC2 highly relevant genes in AtlasEoE, related to Figure 6 The top ILC2 HRGs, along with their relevance and ranks in ILC2s and T_H2 cells, highlight the upregulation of the prostaglandin-related genes *PTGS2* and *PTGDR2*, and the epidermal growth factor family gene *AREG*.

Cell type	Clevenger et al.			Morgan et al.		
	ProtoCloud	CellTypist	scANVI	ProtoCloud	CellTypist	scANVI
Macrophage (*SEPP1*)	905	1607	1061	25	86	67
Macrophage (*SEPP1/IL1B*)	662	357	391	355	321	294
Macrophage (*cycling*)	511	285	311	22	11	13
Macrophage (*PLAC8*)	283	147	501	90	19	53
Macrophage (*ALOX15*)	51	19	81	28	20	74
Macrophage (*SPP1*)	3	1	7	1	10	67
Macrophage (*SEPP1/FOLR2*)	4	1	7	2	1	6
Treg	1679	1967	1983	609	562	821
T_H2	283	266	283	207	202	367
CD4+ Tcm	429	55	171	176	90	75
MAIT (*NCR3*)	209	8	256	45	11	21
T_H1	65	2	55	14	18	7
NK (*FCGR3A*)	45	5	44	9	1	7
ILC2	28	**0**	26	12	1	30
cDC2B (*CD1C*)	1980	1450	2076	179	151	180
Mature DC (*FSCN1*)	563	324	378	28	49	26
cDC1 (*CLEC9A*)	201	141	239	31	24	34
DC (cycling)	240	64	266	3	**0**	3
cDC2A ($IL22RA2^{+}CLEC10A^{-}$)	50	7	43	1	1	2
cDC2C (*PRDM16*)	29	16	23	7	3	10

Table S7: Cell type identification among ProtoCloud, CellTypist, and scANVI across two applied EoE datasets, related to Figure 6 While all models identify common populations, ProtoCloud demonstrates a higher subtype resolution compared to CellTypist. CellTypist shows reduced sensitivity in detecting rare populations, failing to identify ILC2s in the Clevenger dataset and cycling DCs in the Morgan dataset. Although scANVI shows high sensitivity for these rare cell states, some predictions may be of low quality.

References

1. Ding, J., Adiconis, X., Simmons, S. K., Kowalczyk, M. S., Hession, C. C., Marjanovic, N. D., Hughes, T. K., Wadsworth, M. H., Burks, T., Nguyen, L. T., et al. (2020). Systematic comparison of single-cell and single-nucleus RNA-sequencing methods. Nature Biotechnology *38*, 737–746. https://doi.org/https://doi.org/10.1038/s41587-020-0465-8.

2. Tran, N. M., Shekhar, K., Whitney, I. E., Jacobi, A., Benhar, I., Hong, G., Yan, W., Adiconis, X., Arnold, M. E., Lee, J. M., et al. (2019). Single-cell profiles of retinal ganglion cells differing in resilience to injury reveal neuroprotective genes. Neuron *104*, 1039–1055. https://doi.org/10.1016/j.neuron.2019.11.006.

3. Ding, J., Garber, J. J., Uchida, A., Lefkovith, A., Carter, G. T., Vimalathas, P., Canha, L., Dougan, M., Staller, K., Yarze, J., et al. (2024). An esophagus cell atlas reveals dynamic rewiring during active eosinophilic esophagitis and remission. Nature Communications *15*, 3344. https://doi.org/10.1038/s41467-024-47647-0.

4. Clevenger, M. H., Karami, A. L., Carlson, D. A., Kahrilas, P. J., Gonsalves, N., Pandolfino, J. E., Winter, D. R., Whelan, K. A., and Tétreault, M.-P. (2023). Suprabasal cells retain progenitor cell identity programs in eosinophilic esophagitis–driven basal cell hyperplasia. JCI Insight *8*. https://doi.org/10.1172/jci.insight.171765.

5. Morgan, D. M., Ruiter, B., Smith, N. P., Tu, A. A., Monian, B., Stone, B. E., Virk-Hundal, N., Yuan, Q., Shreffler, W. G., and Love, J. C. (2021). Clonally expanded, GPR15-expressing pathogenic effector TH2 cells are associated with eosinophilic esophagitis. Science Immunology *6*, eabi5586. https://doi.org/10.1126/sciimmunol.abi5586.
